# Sail sign in neonatal pneumomediastinum: a case report

**DOI:** 10.1186/s12887-019-1397-7

**Published:** 2019-01-31

**Authors:** Stephen Sze Shing Teo, Archana Priyadarshi, Kathryn Browning Carmo

**Affiliations:** 1 Paediatrics & Emergency Departments, Blacktown & Mt Druitt Hospitals, Sydney, Australia; 2 Paediatrics Department, Western Sydney University, Sydney, Australia; 3 Neonatal Intensive Care Unit, Westmead Hospital, Sydney, Australia; 4 NSW Newborn & Paediatric Emergency Transport Service, Sydney, Australia; 5 Grace Centre for Newborn Intensive Care, Children′s Hospital at Westmead, Sydney, Australia; 6 University of Sydney, Sydney, Australia

**Keywords:** Neonate, Pneumomediastinum, Thymus

## Abstract

**Background:**

Pneumomediastinum is an uncommon cause of neonatal respiratory distress. Clinical history and examination of the neonate may be uninformative in determining the aetiology of the respiratory distress. Chest x-ray can be diagnostic of pneumomediastinum however is often difficult to interpret.

**Case presentation:**

A 36 week gestation newborn was delivered by emergency caesarean section, intubated and given intermittent positive pressure ventilation via Neopuff™ for apnoea before being extubated to CPAP. Chest radiography initially showed bilateral upper zone opacities, presenting a diagnostic dilemma; however subsequent films demonstrated a pneumomediastinum which was managed conservatively and resolved.

**Conclusions:**

The “sail sign” describes an uncommon radiological appearance of a pneumomediastinum in neonates and infants. With careful conservative management, a spontaneous resolution without longterm sequelae can be expected.

## Background

Respiratory distress in the neonate is a frequent clinical scenario. Common causes include transient tachypnoea of the newborn, respiratory distress syndrome, pneumothorax, meconium aspiration syndrome and pneumonia. Pneumomediastinum is uncommon in both infants and children [1, 2]. In the neonate, pneumomediastinum may be asymptomatic but may also cause significant respiratory distress. We present a case of pneumomediastinum in a neonate in whom the initial chest x-rays (CXRs) were difficult to interpret.

## Case presentation

**Fig. 1:**
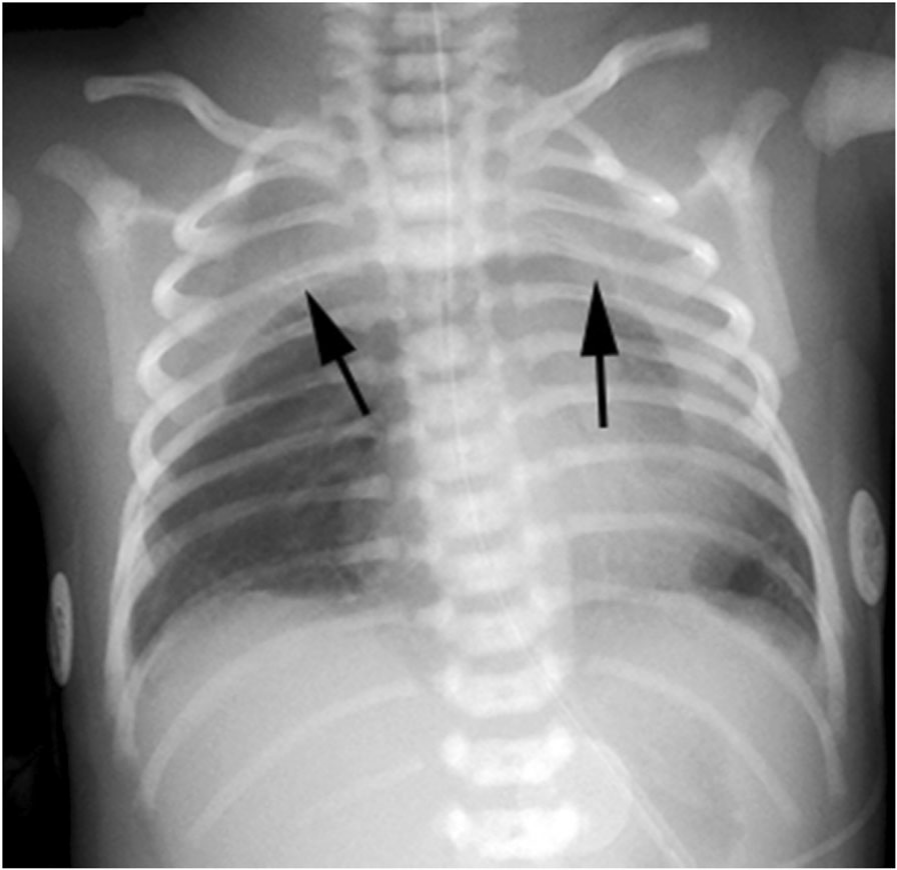
CXR at 2 h of age

We report a male baby born in a peripheral setting at 36 weeks of gestation to a 36 year old primiparous mother whose pregnancy was complicated by breech presentation and premature rupture of membranes at 34 weeks of gestation. Delivery was by emergency caesarean section with maternal general anaesthetic for fetal distress and cord prolapse. Apgar scores were 4, 5, 5, and 8 at one, five, ten and fifteen minutes respectively. He required mask ventilation at birth for primary apnoea and was intubated at seven minutes of postnatal age for persistent apnoea. At 30 min of life he was extubated to CPAP of 5 cmH_2_O and FiO_2_ was weaned from 100 to 44%. On these settings he had a tachypnoea of 90 breaths per minute with mildly increased respiratory effort and a pre-ductal oxygen saturation of ≥97%. A capillary blood gas excluded respiratory or metabolic acidosis. Empiric antibiotics benzylpenicillin and gentamicin were commenced to cover the risk of sepsis due to the prolonged rupture of membranes. Chest x-rays at 2 and 4 h of life showed crescent shaped homogeneous opacities in both upper lung fields (Fig. 1). The Neonatal and Paediatric Emergency Transport Service (NETS) was consulted. Initial paediatric radiological advice via telemedicine was that this appearance could represent bilateral pleural effusions. Accordingly the PEEP was increased to 8cmH_2_O; lateral decubitus x-ray (not shown) was uninformative. Due to the uncertainty regarding the cause of the respiratory distress the baby was transferred to the local neonatal intensive care unit (NICU) by NETS at 10 h of postnatal life.

**Fig. 2:**
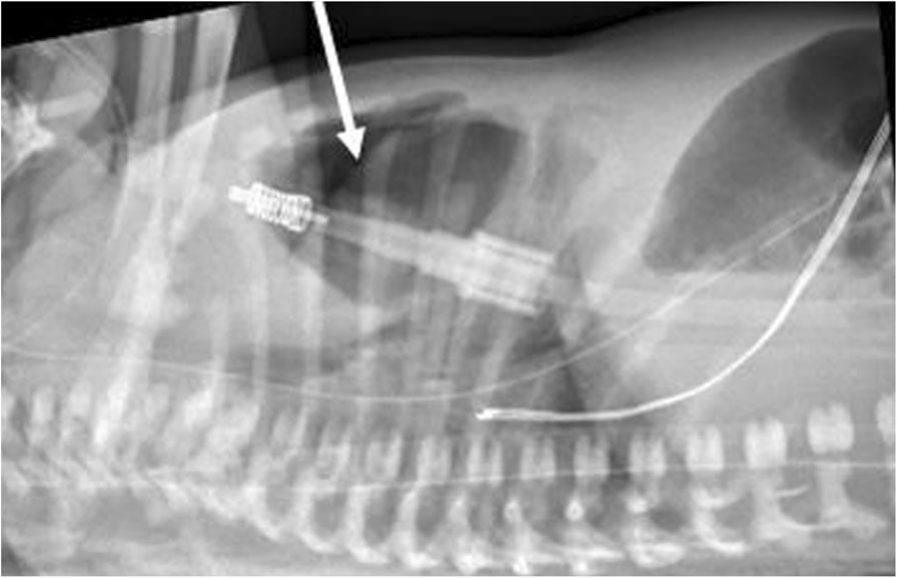
Lateral x-ray demonstrating air anterior to the mediastinum

A progress CXR at just under 13 h of age was still inconclusive. However a lateral decubitus film performed concurrently demonstrated air anterior to the mediastinum, consistent with a diagnosis of pneumomediastinum (Fig. 2); all respiratory support was therefore ceased. Laboratory markers for infection were negative with a white cell count of 15.6 × 10^9^/L and C-reactive protein of < 3.0 mg/L. The clinical examination of the baby was not suggestive of sepsis and chest x-ray findings were not consistent with a diagnosis of congenital pneumonia; thus antibiotics were ceased at 48 h of age. The baby’s respiratory distress resolved without active intervention and progress CXRs demonstrated gradual resolution of the pneumomediastinum. The homogeneous opacity in the upper- and mid-zones that had initially appeared bilateral and then right-sided only, was shown on chest ultrasound to be thymus rather than a mass lesion. A progress CXR at 11 weeks of age and MRI at 4 months of age demonstrated a normal thymus with no other intra-thoracic pathology. The baby was well when discharged from follow-up at 8 months of age.

## Discussion

The incidence of neonatal pneumomediastinum was estimated in the 1960s to be rare at 4–25 per 10,000 live births [1, 2]. The putative pathophysiology of pneumomediastinum was described even earlier. From animal studies in 1939, it was hypothesized that air moves from distended alveoli through very small ruptures between pulmonary capillaries and then flows along perivascular sheaths into the mediastinum, thereby leading to varying degrees of obstruction to pulmonary blood flow and reduction in lung volume [3]. This hypothesis is supported by the reported higher incidence of pneumomediastinum in neonates who have received positive pressure ventilation [4, 5] such as in our patient, although spontaneous cases are also described [2, 5]. Clinical findings if present are those of respiratory distress and there may also be signs due to air leak in other anatomical locations such as a pneumothorax [6].

Diagnosis is usually made on plain chest radiography [7]. CXR may show a halo around the heart border which excludes the diaphragmatic border of the heart, differentiating a pneumomediastinum from a pneumopericardium; or a lateral view may show a retrosternal translucency [8]. In our patient, elevation of the thymus gave rise to a crescentic appearance of the thymus and the “spinnaker-sail sign” [1, 6, 9]. CT scan is the investigation of choice for suspected mediastinal masses [10] and has also been recommended for diagnosis of pneumomediastinum [7], This diagnostic benefit must be weighed against the risks of radiation exposure and an increased long term cancer risk to the newborn [11]. This risk has been attenuated more recently with the development of lower radiation CT strategies [12]. Ultrasound has a far better safety profile and been also been used as an adjunct to CXR in neonates [13, 14].

Although there have been case reports of drainage of a pneumomediastinum in a neonate in extremis [15–17], there is spontaneous resolution reported in case reports and case series in the majority, with or without supplemental oxygen [1, 2, 7, 9, 18].

## Conclusions

Pneumomediastinum is a rare cause of neonatal respiratory distress and initial chest X-rays can be difficult to interpret. Despite impressive findings on chest radiography, management is generally conservative and the prognosis usually excellent.
